# Effects of Resistant Starch Infusion, Solely and Mixed with Xylan or Cellulose, on Gut Microbiota Composition in Ileum-Cannulated Pigs

**DOI:** 10.3390/microorganisms12020356

**Published:** 2024-02-09

**Authors:** Yaowen Zhang, Yu Bai, Zhenyu Wang, Hao Ye, Dandan Han, Jinbiao Zhao, Junjun Wang, Defa Li

**Affiliations:** 1 State Key Laboratory of Animal Nutrition and Feeding, College of Animal Science and Technology, China Agricultural University, Beijing 100193, China; 2 Animal Nutrition Group, Department of Animal Sciences, Wageningen University and Research, 6708 PB Wageningen, The Netherlands; 3 Adaptation Physiology Group, Department of Animal Sciences, Wageningen University and Research, 6708 PB Wageningen, The Netherlands

**Keywords:** resistant starch, xylan, cellulose, gut microbiota, SCFA, large intestine fermentation, ileum-cannulated pigs

## Abstract

Fermentation of dietary fiber (DF) is beneficial for gut health, but its prebiotic effects are often impeded in the distal large intestine because of the fast degradation of fermentable substrates. One way to enhance the prebiotic effect of DF is to deliver fibers to the lower parts of the gut, which can be achieved by mixing different kinds of fiber. Therefore, in the present study, an ileum-cannulated pig model was employed to investigate the fermentation influence in the large intestine by infusing resistant starch solely (RS, fast fermentable fiber) and mixing with other fibers (xylan or cellulose). Twenty-four ileum-cannulated growing pigs were divided into four groups: one control group receiving saline ileal infusions and three experimental groups infused with RS, RS with xylan, or RS with cellulose. Fecal and plasma samples were analyzed for gut microbiota composition, short-chain fatty acids (SCFAs), and blood biochemistry. Results indicated no significant differences between the RS and control group for the microbiome and SCFA concentration (*p* > 0.05). However, RS combined with fibers, particularly xylan, resulted in enhanced and prolonged fermentation, marked by an increase in *Blautia* and higher lactate and acetate production (*p* < 0.05). In contrast, RS with cellulose infusion enriched bacterial diversity in feces (*p* < 0.05). Blood biochemistry parameters showed no significant differences across groups (*p* > 0.05), though a trend of increased glucose levels was noted in the treatment groups (*p* < 0.1). Overall, RS alone had a limited impact on the distal hindgut microbiota due to rapid fermentation in the proximal gut, whereas combining RS with other fibers notably improved gut microecology by extending the fermentation process.

## 1. Introduction

Emerging research highlights the significant role of gut microbiota and their fermentation products in animal gut health. Dietary fibers (DF) are noted for their positive physiological effects, mediated via intestinal microbiota activity [1]. Moderate DF consumption is linked to numerous health benefits [1,2], particularly in enhancing gut ecology by boosting populations of beneficial bacteria and increasing short-chain fatty acid (SCFA) production [3]. SCFA are vital for colonic health, serving as a key energy source for colon cells and maintaining colonic mucosa integrity [4].

Identifying DF types that enhance beneficial fermentation in the large intestine is a crucial research focus. Resistant starch (RS), defined as a type of DF, encompasses all starch that are not absorbed in the small intestine of healthy individuals. RS is categorized into five types based on their physical and chemical properties: physically entrapped starch (RS1), ungelatinized starch granules (RS2), retrograded starch (RS3), chemically modified starch (RS4), and amylose-lipid complexes (RS5) [5]. Among these types, RS3 is particularly noteworthy due to its safety and thermal stability when added as an ingredient to processed foods [6]. Like other types of RS, RS3 resists enzymatic degradation in the foregut and undergoes fermentation in the hindgut. This process promotes the growth of beneficial microbes and the production of SCFA, contributing to microbial balance and intestinal health [7].

Nevertheless, the prebiotic impact of DF has its constraints. Fast fermenting DFs, such as RS, often undergo complete degradation in the cecum and fail to extend to the distal colon [8,9], thereby diminishing their prebiotic advantages [10]. Inadequate fermentation of fibers in the distal hindgut may compromise the colonic mucus barrier, foster harmful bacteria growth, and increase vulnerability to pathogens [11]. Therefore, it is vital to explore methods for delivering fibers to the distal colon more effectively, ensuring a sustained metabolite production and energy provision for this region, while fostering beneficial bacterial growth [12]. Research has shown that the fermentation site of fiber in the large intestine is influenced by the presence of other fibers [13]. Another study suggested that blending a rapidly fermentable fiber like RS with a complex, slower fermenting fiber could satisfy the microbiota in the proximal gut. This might enhance the amount of slowly fermentable fiber reaching the distal colon [9]. Thus, using a blend of fibers could offset the limitations of fast-fermenting fibers, ensuring extended fermentation and sustain prebiotic function [14].

Cellulose and hemicellulose, specifically xylan, constitute significant portions of fermentable DF and exhibit notable structural disparities. Our prior investigations have explored the distinct fermentation profiles of xylan and cellulose through pigs and in vitro experiments, highlighting variations in their fermentation characteristics [15,16,17]. Utilizing a fiber-free basal diet for examining the fermentation metabolism of individual fiber components is advantageous. This approach allows for a deeper understanding of the effects of specific complex carbohydrates on the gastrointestinal tract of animals, without interference from other fibers. Moreover, employing ileum-cannulated pigs as a model enables precise evaluation of the isolated fermentation effects of fiber fractions in different gut segments, while avoiding confounders like gastric juices and bile, thus facilitating accurate assessment of intestinal responses to various fiber substances.

Therefore, in the present study, fermentation of substrates in the distal gut, without modifications in the foregut, was observed by infusing RS alone, and in combination with xylan or cellulose, into ileum-cannulated pigs. The objective of this study was to validate the efficacy of mixed fermentation as a method to enhance the fermentation of fibers in the distal hindgut. It was hypothesized that the mixture of RS with other fibers would extend prebiotic benefits beyond solitary infusion, thereby improving gut microecology in growing pigs.

## 2. Materials and Methods

### 2.1. Experimental

This study followed the Laboratory Animal Welfare and Animal Experimental Ethical Inspection Committee in China Agricultural University (AW12601202-1-1) and was approved by the Welfare and Ethics Committee of the Chinese Association for Laboratory Animal Sciences.

#### 2.1.1. DF Material

Purified RS3 powder was supplied from Jiechun Biotechnology Co., Ltd. (Shanghai, China), which was all derived from corn and produced by heat-moisture treatment. The RS used in this study was insoluble (soluble percentage *: 1.7 ± 0.3). Xylan (soluble) and cellulose (insoluble) were purchased from the Pioneer biotech company (Xi’an, China).

* The solubility test for RS was performed as follows: 5 g of RS was mixed with 100 mL of saline and continuously stirred at 39 °C for 1 h. Afterward, the mixture underwent centrifugation at 3000× *g* for 10 min. The supernatant was then decanted, and the residual sample was dried using a stream of nitrogen gas. The dry sample was subsequently weighed. This test was conducted with three replicates. The percentage of soluble RS was calculated as follows:

$$\mathrm{Percentage}\;\mathrm{of}\;\mathrm{Soluble}\;\mathrm{RS}=\left(\frac{\mathrm{original}\;\mathrm{weight}\;\mathrm{of}\;\mathrm{RS}-\mathrm{Weight}\;\mathrm{of}\;\mathrm{residue}\;\mathrm{after}\;\mathrm{dring}}{\mathrm{Original}\;\mathrm{weight}\;\mathrm{of}\;\mathrm{RS}}\right)\times 100$$

#### 2.1.2. Animals

Experiments were conducted at the FengNing Swine Research Unit of China Agricultural University (Academician Workstation in Chengdejiuyun Agricultural and Livestock Co., Ltd., Chengde, Hebei, China). Twenty-four healthy growing pigs (68–70 days of age, Duroc × Landrace × Large White, half male and half female) with 25 ± 1.69 kg (mean ± SE) weight were used in this experiment. They were individually housed in stainless metabolism crates. After 7 d acclimation period, pigs were fasted for 12 h prior to fitting a simple T-cannula in the distal ileum, approximately 5 cm cranial to the ileocecal sphincter. The surgery operation details have been described previously [18]. Then, the pigs were gradually adapted to standard corn-soybean meal diet for recovering. After the 21 d recovery period, pigs were assigned into four groups with six replicates in each group. The pigs in the these groups were subjected to infusions as follows: 50 mL of sterile saline (Con, blank control), 50 mL of RS suspension (30 g RS3 suspended in 50 mL of sterile saline; RS), 50 mL of a mixture of RS and xylan suspension (15 g of RS3 and 15 g xylan mixture suspended in 50 mL of sterile saline, RX) and 50 mL of a mixture of RS and cellulose suspension (15 g of RS3 and 15 g cellulose mixture suspended in 50 mL of sterile saline, RC). All the experimental pigs were fed with same fiber-free diet to prevent other fiber interference and keep the infusion of fiber as the only variation. The diet was formulated to exceed the nutrients requirement of NRC (2012) and the nutrients composition was listed in Supplementary Table S1. Diet was divided into two equivalent daily meals and provided at 08:30 and 16:30; the mixtures of saline with different dietary fibers were infused, respectively, after 30 min. The whole experiment lasted for 21 d.

#### 2.1.3. Sample Collection

At the end of the experiment (Day 21), pig’s feces were collected from the rectum into 2 mL centrifuge tubes and frozen in liquid nitrogen and stored at −80 °C for microbiota genes and SCFA analysis. Blood samples (8 mL) were intravenously collected 2 h postprandially in EDTA tubes from the anterior vena cava of the experimental pigs and sera were collected by 3000× *g*, 10 min centrifugation for biochemistry analyses.

### 2.2. 16S rRNA Sequence Processing and Bioinformatics Analyses

DNA in feces were extracted using a FastDNA SPIN Kit for soil (MP Biomedicals, Irvine, CA, USA) according to manufacturer’s protocol. Briefly, fecal samples were homogenized in a lysis solution, followed by mechanical disruption using a bead beater. The lysate was then subjected to a series of washes and spins to purify the DNA, which was eluted in a final buffer. Extracted DNA was quantified using NanoDrop 2000 (Thermo Fisher Scientific, Waltham, MA, USA). The ratio of absorbance at 260 nm and 280 nm was calculated to evaluate the purity of the DNA.

Bacteria 16S rRNA sequencing genes (V3-V4 regions) were amplified with primer pairs 338F (5′-ACTCCTACGGGAGGCAGCAG-3′) and 806R (5′-GGACTACHVGGGTWTCTAAT-3′). PCR reactions were carried out in a 25 μL mixture containing 12.5 μL of PCR Master Mix, 1 μL of each primer (10 μM), 2 μL of DNA template, and 8.5 μL of nuclease-free water. Amplification was performed in an ABI GeneAmp 9700 PCR thermocycler (ABI, Los Angeles, CA, USA). The optimized conditions for PCR amplification were as follows: initial denaturation at 95 °C for 3 min, 27 cycles of denaturing at 95 °C for 30 s, annealing at 55 °C for 30 s and extension at 72 °C for 45 s, followed by a final extension at 72 °C for 10 min. PCR products were verified via agarose gel electrophoresis and purified using a Gel Extraction Kit (Qiagen, Hilden, Germany). The purified amplicons were quantified using the Qubit dsDNA HS Assay Kit (Thermo Fisher Scientific, USA). The purified amplicons were pooled in equimolar ratios and prepared for sequencing using the Illumina MiSeq PE300 platform (Illumina, San Diego, CA, USA) according to the standard protocols by Majorbio Bio-Pharm Technology Co., Ltd. (Shanghai, China). The library preparation for sequencing involved the attachment of sequencing adapters and indices using the Illumina TruSeq DNA LT Sample Prep Kit. The FASTQ files with valid data were submitted to the National Center for Biotechnology Information (NCBI) Sequence Read Archive (SRA) under the accession numbers PRJNA1057771.

The 16S raw sequencing reads were demultiplexed according to sample-specific barcode (6–8 nucleotides) and imported in QIIME2 platform (version 2020.2). Each sample’s reads were separated and quality checked. The demultiplexed reads underwent quality control and denoising in QIIME2 using the DADA2 plugin with default parameters. This process involved trimming and filtering the reads, modeling and correcting errors in the reads, and merging the paired end reads. Post-denoising, ASVs were generated. Only ASVs with minimum abundance of two reads and detected in more than two samples were retained. All ASVs were classified against silva 132 database by naïve bayes classifier constructed by scikit-learn, Qiime2 software (version 2020.2). The α-and β-diversity were calculated using vegan package (version 2.5-6) inside R. The α-diversity indices included observed species, Shannon diversity, and Chao1 richness. The β-diversity was assessed using Bray–Curtis dissimilarity, and Principal Coordinates Analysis (PCoA) was performed to visualize the differences in microbial communities across samples. Differential abundance of microbial genera among different groups was identified using the Linear Discriminant Analysis Effect Size (LEfSe) method. This analysis helped to pinpoint specific bacterial taxa that were significantly different in abundance between the study groups.

### 2.3. Quantification of SCFA

The SCFA concentration from feces was determined according to the published method [19]. Briefly, 0.5 g–1 g sample was added to 8 mL deionized water. The mixture was thoroughly homogenized by vortexing and centrifuged at 13,000× *g* for 10 min. Then, diluted the supernatant (50 times for large intestine) and filtered through a 0.22-μm filter (Millipore, Bedford, OH, USA). Samples (25 μL) were analyzed using a Dionex ICS-3000 high-performance ion chromatograph (Thermo Fisher Scientific, Waltham, MA, USA) equipped with an Acclaim Organic Acid OA column. Potassium hydroxide (KOH) was employed as the mobile phase in a gradient mode, maintaining a flow rate of 0.5 mL/min. Acids were identified through conductivity detection after eluent suppression. Quantification was achieved using calibration curves of lactic, acetic, propionic, and butyric acids.

### 2.4. Blood Biochemistry Analyses

The plasma was analyzed for glucose (Glucose Kit), TG (Triglycerides Kit), TC (Total Cholesterol Kit), HDL (Direct High-Density Lipoprotein Cholesterol Kit), LDL (Direct Low-Density Lipoprotein Cholesterol Kit), TP (Total Protein Kit), ALB (Albumin Kit), and GLB (Globulin Kit); the colorimetry determination was operated using an automatic biochemical analyzer (BS-420, Mindary Biomedical Electronics Co., Ltd., Shenzhen, China) based on the manufacturer’s instructions, which were all procured from BioSino Bio—Technology & Science Inc. (Beijing, China).

### 2.5. Statistical Analyses

The data were presented as means of six replicates with standard deviations. Analysis of variance (ANOVA) using Tukey’s test was performed with SPSS statistical software (version 20.0, SPSS Inc., Chicago, IL, USA). Significant differences of the mean values were determined at *p* < 0.05 and statistical trends were defined as *p* < 0.1. QIIME2 and R package software (v3.2.0) was used for the statistical analysis of microbiota bioinformatics.

## 3. Results

### 3.1. Fermentation Effects on Gut Microbiota

#### 3.1.1. Microbiome Diversity

**Figure 1:**
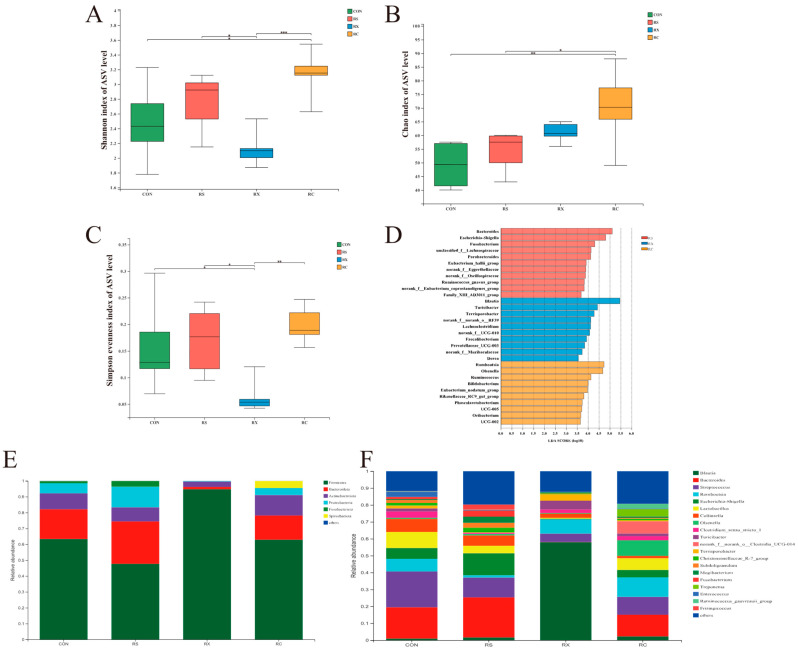
Microbiota diversity and composition of fecal samples. Shannon index (**A**), Chao index (**B**), and Simpson evenness index (**C**). The results were analyzed by the Kruskal–Wallis H test and presented as mean values, * *p* < 0.05, ** *p* < 0.01, *** *p* < 0.001; LEfSe bar; (**D**) shows the most differential taxa among groups at the genus level. The mean relative abundance of top 6 phylum along the gastrointestinal tract (**E**). The mean relative abundance of top 20 genus along the gastrointestinal tract (**F**), *n* = 6.

The alterations in microbiota serve as crucial indicators of the fermentation of dietary fiber within the intestinal tract. To examine the impact of a mixture of resistant starch (RS) combined with xylan or cellulose on the microbial community residing in the large intestine of growing pigs, 16S sequencing was conducted on their fecal samples. Microbiome diversity was assessed at the amplicon sequence variant (ASV) level utilizing the Shannon diversity index, the Chao index, and the Simpson evenness index, which evaluate various facets of diversity, encompassing species richness, abundance, and evenness among experimental groups (Figure 1). All three indices exhibited concordant trends, wherein the RX group displayed the lowest levels of species diversity, abundance, and evenness among all experimental groups, significantly diverging from both RS and RC groups (*p* < 0.05). Conversely, the RC group exhibited the highest indices across all groups, signifying an enhancement in species diversity resulting from cellulose fermentation. No significant disparities were observed between the RS and control groups (*p* > 0.05).

#### 3.1.2. Microbiome Composition and Linear Discriminant Analysis Effect Size

The relative bacterial abundances across distinct experimental cohorts were depicted at both phylum and genus levels (Figure 1E,F). The fecal samples from control and experimental groups exhibited the presence of primarily six different phyla, namely Firmicutes, Bacteroidota, Actinobacteriota, Proteobacteria, Fusobacteriota, and Spirochaetota. Among these, Firmicutes, followed by Bacteroidetes, Actinobacteria, and Proteobacteria, predominated in all groups. Notably, the RS-infused group displayed a heightened relative abundance of Bacteroidota, the abundance of Firmicutes increased in the RX group, and Spirochaetota in the RC group compared to the control group (Figure 1E).

At the genus level, members of genera *Blautia*, *Bacteroides*, *Streptococcus*, *Romboutsia*, *Escherichia-Shigella* and *Lactobacillus* dominated the feces samples, although distinct variations existed within these groups. *Bacteroides* and *Escherichia-Shigella* were the most predominant genus in the RS group. Meanwhile, the infusion of RX led to a notable increase in the abundance of *Blautia*, which accounted for over 50% of the total bacterial population in the fecal samples. However, in the RC group, no singular dominant genus was discernible (Figure 1F).

Linear discriminant analysis effect size (LEfSe) confirmed the previously observed results (Figure 1D). The top two discriminating genera for pigs only infused with RS were *Bacteroides* and *Escherichia-Shigella*. The genus of *Blautia* and *Turicibacter* emerged as the distinguishing genera for RX group, while the *Romboutsia* and *Olsenella* were found to be characteristic of the RC group.

#### 3.1.3. Principle Coordinate Analysis

**Figure 2:**
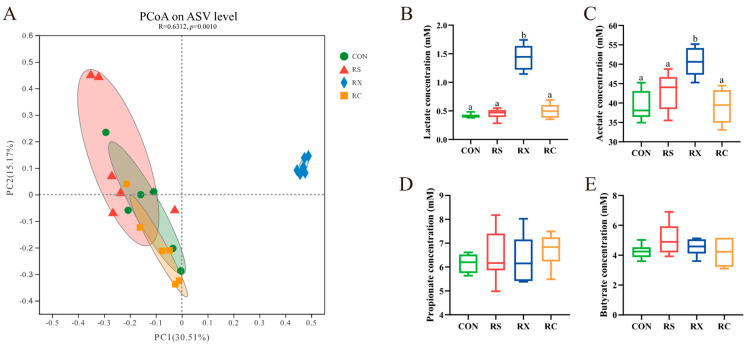
Principle Coordinate Analysis and SCFA concentration. PCoA plots (**A**) under Bray−Curtis distance metrics on ASV level, each symbol represents a fecal sample of one group (*n* = 6). Quantification of lactate (**B**), acetate (**C**), propionate (**D**), and butyrate (**E**) in the feces of different groups. Data were analyzed by the one−way ANOVA with Tukey’s post −hoc test and presented as the mean values (*n* = 6). Different letters mean *p* < 0.05.

The Principle Coordinate Analysis (PCoA) was performed to meticulously assess the microbiomes of the individual subjects included in this study (Figure 2A). A notable differentiation was observed, with the RX group displaying a significant separation from the other experimental groups (*p* < 0.05), while pigs infused solely with RS or those receiving RS mixed with cellulose exhibited closer clustering.

### 3.2. Quantification of SCFA

Fecal SCFAs concentrations were quantified to evaluate the fermentative capacity of intestinal microbiota in response to dietary fiber (Figure 2). Within the RX group, pigs exhibited significantly elevated levels of lactate and acetate in fecal samples when compared to the other groups (*p* < 0.05). For the propionate and butyrate concentration, insignificant difference was observed among all four groups (*p* > 0.05).

### 3.3. Blood Biochemistry Analyses

The postprandial venous blood was collected for blood biochemistry analysis. As shown in Table 1, insignificant differences were observed in the postprandial plasma glucose, protein, and lipids indexes when pigs were infused with RS and other fibers (*p* > 0.05). This is probably because the energy absorption was much lower in the hindgut, thus the infusion of fibers could not affect the blood indexes. However, there is an improvement tendency for glucose concentration in the three DF infusion groups compared with the control group (*p* < 0.1).

## 4. Discussion

Dietary fibers contribute to health and physiology primarily via the fermentative actions of the host’s gut microbiome. RS has demonstrated its fermentation benefits in pig production in numerous studies [20,21,22]. However, the prebiotic effects of RS are constrained by its rapid fermentation, with complete degradation occurring in the cecum and proximal colon [23]. Additionally, research has indicated that bacteria in the small intestine could also affect RS degradation [24]. Therefore, in this experiment, the utilization of the ileum-cannulated pig model and a diet devoid of dietary fibers were employed to mitigate the impact of small intestine bacteria. Both soluble and insoluble fibers (xylan and cellulose) were separately incorporated with RS for infusion into growing pigs. The objective of this methodology is to facilitate the transportation of fibers to the more distal segments of the large intestine, thereby resulting in extended prebiotic fermentation benefits and an enhancement in the composition of the gut microbiota.

The corn RS3 was used in our experiment. A previous study from our laboratory fed growing pigs with the same RS3 to investigate the dietary effects on microbiome of different gut segments [15]. We found that the RS were mostly fermented in the ileum and cecum of pigs and contributed the increasing abundance of *Bacterodes*, *Escherichia-Shigella* and *Turicibacter*, indicating these bacteria possess the capacity to utilize RS as fermentation substrates [15]. Our current study partially reaffirmed these conclusions. Specifically, we observed that the infusion of RS3 led to a slight increase in the abundance of *Bacteroidota* at the phylum level and *Bacteroides* at the genus level in fecal samples. Additionally, the LEfSe analysis identified *Escherichia-Shigella* as occupying the second position in terms of significance. However, the improvements observed were constrained in comparison to the fecal samples of the control group. Additionally, no noteworthy changes were observed in the abundance of Turicibacter when compared to the control group. These results may be attributed to the complete utilization of RS in the proximal large intestine, as supported by our previous research where the abundance of *Turicibacter* decreased from the cecum to the feces [15]. The result from PCoA also confirmed this for the clustering distribution of the RS and CON group.

Conversely, the microbiota structures were apparently changed upon the infusion of a mixture of RS and xylan. The relative abundance of *Blautia* boosted substantially at genus level, which also contributed to the increasing abundance of Firmicutes at the Phylum level [25]. These findings are consistent with our laboratory’s earlier study, where feeding the same xylan to growing pigs significantly increased the abundance of *Blautia* in the cecum and colon [15]. However, its abundance decreased from the proximal colon, comprising only a minor percentage in the fecal samples of the previous experiment, which means the xylan was fermented out before it reached the distal hindgut. In contrast, our current study revealed that more than half of the abundance of *Blautia* was found in the feces, indicating the presence of RS prolonged the fermentation of xylan, allowing its fermentative effects to persist into the terminal rectum. *Blautia* has garnered particular attention due to its potential role in alleviating inflammatory and metabolic diseases, as well as its antibacterial properties [25,26]. The abundance of this bacterium is predominantly influenced by dietary factors, and some studies have unveiled a positive correlation between the proliferation of *Blautia* and the consumption of DF [27,28,29]. By feeding the same xylan to growing pigs, our previous study showed an increased abundance of *Bifidobacterium* [15], which was not observed in RX group in the present experiment. The observed inconsistency is intriguing, as the rising abundance of *Blautia* suggests that xylan was still undergoing fermentation in the distal intestine. This phenomenon could be attributed to the presence of RS, which might have influenced the utilization of bacteria for xylan, potentially contributing to the growth of *Blautia*, given that all *Blautia* strains are capable of utilizing glucose [25]. The genus of *Turicibacter* was shown to be one of the dominant genera in the RX group by LEfSe analysis (Figure 1D). The abundance of this genus was also observed in the large intestines of pigs that were fed the same RS and xylan, individually [15]. In this study, the *Turicibacter* may be promoted by the mixture of RS and xylan. The dominance of *Blautia* also provides an explanation for the reduced community diversity observed in the feces of the RX group. This observation is consistent with our previous findings and other reports; they proved that xylan supplementation resulted in lower observed features and a decreased Shannon index in the hindgut of pigs [15,25]. Consistent with the α-diversity, distinct separation was observed in the community composition between RX and the other groups for PCoA. This difference could be attributed to the increasing abundance of *Blautia* as well. Overall, the changes by infusing a mixture of RS and xylan were mainly induced by xylan fermentation. Jonathan et al. reported that RS could delay the fermentation of other dietary fibers in the hindgut of pigs by redirecting microbial metabolism toward the utilization of starch [29]. Therefore, only xylan substrates remained available for fermentation in the distal part of the intestine, which offers an explanation as to why the fecal microbiome and SCFA concentration became xylan-fermentation oriented.

Fecal samples from pigs of the RC group exhibited the highest community diversity relative to the other groups. This outcome supports the conclusions drawn from our prior experiment [17], which involved infusing the mixture of the same cellulose and digestible corn starch into growing pigs, revealing a significant increase in the Shannon index of fecal microbiota due to the inclusion of cellulose. The microbiota composition of the RC group exhibited a close resemblance to that of the RS and CON groups in the current experiment. However, in contrast to the RS group, the RC group did not exhibit a clearly dominant genus. The result of the LEfSe showed that *Romboutsia* and *Olsenella* were the discriminating genera which is inconsistent with our prior in vitro fermentation findings in our laboratory [16] but is partially in line with the outcomes of the sole infusion of digestible starch [17]. In addition to the distinctions between in vitro and in vivo conditions, as well as variations among individual subjects, it is possible that this outcome was attributed to the incomplete fermentation of cellulose. Future research, considering fermentation rate and dosage of DF, will need to be conducted.

The variation in fermentation characteristics among these groups was also reflected in the concentration of SCFA in the fecal samples. The groups with close community diversity (CON, RS and RC) all exhibited similar SCFA levels. Only infusion of a mixture of RS and xylan showed a significant increasing concentration of lactate and acetate acid compared with other groups. This change may be attributed to the improving *Blautia* genus because the final products of glucose fermentation by *Blautia* are acetic acid, succinic acid, lactic acid, and ethanol [25]. One of the advantageous outcomes of fiber fermentation is the production of SCFA within the gastrointestinal tract. SCFA plays a pivotal role in maintaining colonic homeostasis and is intricately involved in regulating immune functions [30,31,32]. Hence, the fermentation of a mixture of RS and xylan resulted in an enhanced gut environment in pigs by increasing the SCFA production.

In our investigation into the impact of fibers infusion on postprandial blood markers in growing pigs, a wide range of blood biochemical indicators were analyzed, including plasma glucose, protein, and lipids. However, the analysis revealed no significant differences for the fibers’ infusion in these blood biochemistry parameters. This could be attributed to the limited impact of pure fermentation in the hindgut on blood parameters such as protein, although a trend toward increased glucose levels was noted in the treated groups. Hosts primarily derive energy from carbohydrates in the small intestine, where starch undergoes enzymatic breakdown into glucose before intestinal absorption [22]. In contrast, the energy derived from the fermentation products, particularly SCFA, resulting from fiber fermentation in the hindgut, is crucial for maintaining the gut ecosystem and overall health in swine [33]. It is estimated that these fermentation products can provide approximately 15% of the maintenance energy needs for growing pigs [34]. Generally, the reduction in digestible energy resulting from RS feeding can be partially offset by the increase in fermentative energy production [20]. In this study, the fermentable carbohydrate substrates were increased in the hindgut of pigs without influencing foregut digestion. This likely explains the observed rising trend in blood glucose concentrations among pigs in the RS, RX, and RC groups.

## 5. Conclusions

This study compared the effects of RS fermentation and its combination with xylan or cellulose, by infusing them into the terminal ileum of growing pigs. In the RS infusion group, the fast fermentation of RS led to no discernible difference in its impact on fecal samples, which means the sole infusion of RS did not sustain the fermentation of fibers at the distal gut. In contrast to the RS group, the mixed infusion effectively extended the fermentation of fibers at the end of the intestine, manifested by the improved microbiota composition and diversity in the fecal samples of the RX and RC group. Especially the mix of RS and xylan increased the levels of beneficial bacteria (*Blautia*, etc.) and SCFA in the distal large intestine. These findings indicate that the strategic combination of different fibers can effectively enhance the gut microecology, particularly in the distal hindgut. It provides a valuable basis and practical insight for optimizing fiber utilization and maximizing their prebiotic effects in the gut of pigs. Further studies are needed to explore the combination of more fiber types particularly focusing on their synergistic effects, and to observe their long-term impacts on the gut ecosystem and overall health outcomes.

## Figures and Tables

**Table 1:** Variations in blood biochemical parameters of pigs after infusion.

Sample	CON	RS	RX	RC	*p*-Value
GLU (mmol/L)	6.56 ± 0.28	7.07 ± 0.42	6.91 ± 0.25	6.90 ± 0.36	0.091
TG (mmol/L)	1.33 ± 0.19	1.41 ± 0.25	1.53 ± 0.12	1.53 ± 0.19	0.235
TC (mmol/L)	2.26 ± 0.22	2.36 ± 0.32	2.31 ± 0.17	2.45 ± 0.16	0.517
HDL (mmol/L)	1.41 ± 0.09	1.50 ± 0.11	1.40 ± 0.08	1.54 ± 0.20	0.174
LDL (mmol/L)	1.43 ± 0.20	1.45 ± 0.22	1.45 ± 0.13	1.57 ± 0.13	0.478
TP (g/L)	58.24 ± 2.69	60.39 ± 3.06	60.16 ± 1.82	61.67 ± 1.48	0.123
ALB (g/L)	22.45 ± 2.06	23.17 ± 3.13	22.03 ± 1.81	22.41 ± 2.26	0.868
GLB (g/L)	36.39 ± 2.46	37.93 ± 1.61	37.95 ± 1.40	36.84 ± 1.47	0.350

Results are expressed as mean ± standard deviation; values represent the average of duplicate analyses from different groups (*n* = 6). GLU, glucose; TG, triglycerides; TC, total cholesterol; HDL, high-density lipoprotein; LDL, low-density lipoprotein; TP, total protein; ALB, albumin; GLB, globulin; CON, control group; RS, resistant starch group; RX, resistant starch and xylan group; RC, resistant starch and cellulose group.

## Data Availability

Data are contained within the article.

## Supplementary Materials

The following supporting information can be downloaded at: https://www.mdpi.com/article/10.3390/microorganisms12020356/s1; Table S1: Ingredient composition of fiber-free diet.
