# Supplementary material for: Effects of Resistant Starch Infusion, Solely and Mixed with Xylan or Cellulose, on Gut Microbiota Composition in Ileum-Cannulated Pigs
Source: Microorganisms. 2024 Feb 9;12(2):356. doi: 10.3390/microorganisms12020356 (PMC10893309; doi:10.3390/microorganisms12020356)
Supplement: Supplementary file 1 [file microorganisms-12-00356-s001.zip › microorganisms-2854472-supplementary.pdf]

Supplementary data

Supplementary Table S1. Ingredient composition of fiber-free diet

Items (%)	Content
Corn starch	61.0
Soybean isolated protein	19.5
Soybean oil	3.3
Sucrose	11.2
Lysine	0.4
Met	0.1
Thr	0.2
Limestone	0.5
Dicalcium Phosphate	2.5
Cr2O3	0.3
Salt	0.5
Premix[1]	0.5
Total	100.0

[1]Premix provided the following per kg of complete diet for growing pigs: vitamin A, 5,512 IU; vitamin D3, 2,200 IU; vitamin E, 64 IU; vitamin K3, 2.2 mg; vitamin B12, 27.6 ug; riboflavin, 5.5 mg; pantothenic acid, 13.8 mg; niacin, 30.3 mg; choline chloride, 551 mg; Mn, 40 mg; Fe, 100 mg; Zn, 100 mg; Cu, 100 mg; I, 0.3 mg; Se, 0.3 mg; Met, DL-Methionine; Thr, L-Threonine.
